# Piperine Ameliorates Trimellitic Anhydride-Induced Atopic Dermatitis-Like Symptoms by Suppressing Th2-Mediated Immune Responses via Inhibition of STAT6 Phosphorylation

**DOI:** 10.3390/molecules25092186

**Published:** 2020-05-07

**Authors:** Dae Woon Choi, Sun Young Jung, Dong-Hwa Shon, Hee Soon Shin

**Affiliations:** 1 Food Biotechnology Program, Korea University of Science and Technology, Daejeon 34113, Korea; choidw19@gmail.com (D.W.C.); 50014@kfri.re.kr (S.Y.J.); 2 Division of Functional Food Research, Korea Food Research Institute, 245, Nongsaengmyeong-ro, Iseo-myeon, Wanju-gun, Jeollabuk-do 55365, Korea; 3 Department of Food Processing and Distribution, Gangneung-Wonju National University, Gangneung, Gangwon-do 25457, Korea; dhs95@gwnu.ac.kr

**Keywords:** piperine, atopic dermatitis, Th2 immune response, CCR3, STAT6

## Abstract

Atopic dermatitis (AD) is a common inflammatory skin disease predominately related to Type 2 helper T (Th2) immune responses. In this study, we investigated whether piperine is able to improve AD symptoms using a trimellitic anhydride (TMA)-induced AD-like mouse model. Topical treatment with piperine reduced ear swelling (ear thickness and epidermal thickness) induced by TMA exposure. Furthermore, piperine inhibited pro-inflammatory cytokines such as TNF-α and IL-1β in mouse ears, compared with the TMA-induced AD group. In measuring allergic immune responses in draining lymph nodes (dLNs), we found that IL-4 secretion, GATA3 mRNA level, and STAT6 phosphorylation were suppressed by piperine treatment. In an ex vivo study, piperine also inhibited the phosphorylation of STAT6 on the CD4^+^ T cells isolated from splenocytes of BALB/c mice, and piperine suppressed IL-4-induced CCL26 mRNA expression and STAT6 phosphorylation in human keratinocytes resulting in the inhibition of infiltration of CCR3^+^ cells into inflammatory lesions. These results demonstrate that piperine could ameliorate AD symptoms through suppression of Th2-mediated immune responses, including the STAT6/GATA3/IL-4 signaling pathway. Therefore, we suggest that piperine is an excellent candidate as an inhibitor of STAT6 and may help to improve AD symptoms.

## 1. Introduction

Atopic dermatitis (AD) is a common inflammatory skin disease that has increased in prevalence over the past decades. The fundamental cause of AD is not completely understood, but its pathogenesis has been associated with immune dysfunction. From previous studies, AD patients predominantly show Type 2 helper T2(Th2) inflammatory responses. Eighty-five percent of AD occur before the age of 5 years, and pediatric patients show a Th1/Th2 imbalance with a predominant Th2 immune response [1,2].

Naïve CD4^+^ T helper cells differentiate into functionally distinct subsets of effector T helper cells by recognizing the antigen presented by the “antigen-presenting cells” (APC) via the T cell receptor complex [3,4]. Among the subsets of effector T cells, Th1 cells are developed upon exposure to interleukin (IL)-12 and secrete interferon (IFN)-γ. Th2 cells produce Th2 cytokines, such as IL-4, IL-5, and IL-13, which induce immunoglobulin (Ig) E synthesis by B cells by mediating isotype switching [5,6]. IL-4 is critical for the differentiation of naïve CD4^+^ T cells into Th2 cells. Signal transducer and activator of transcription 6 (STAT6) has been known to be a transcription factor of Th2 cells. IL-4 induces activation of the STAT6 signaling pathway via phosphorylation of JAK1 and JAK3 [7,8,9,10,11].

Chemokines are small proteins that regulate leukocyte trafficking. Chemokine (C-C motif) ligand 26 (CCL26)/eotaxin-3 is a member of the eotaxin subfamily, which includes CCL11/eotaxin-1, CCL24/eotaxin-2, and CCL26/eotaxin-3 [12,13]. IL-5 causes maturation of the eosinophils from bone marrow-derived CD34^+^ precursor cells and expression of CCR3, which plays a crucial role in the infiltration of eosinophils [14,15,16]. CCL26 is a ligand of CC chemokine receptor 3 (CCR3) and induces chemotaxis of CCR3-expressing cells. CCR3 is predominantly expressed on eosinophils, which play a critical role in allergic diseases, such as AD, allergic rhinitis, and asthma [17]. Th2 cytokines have been known to elicit CCL26 production in keratinocytes through induction of STAT6 phosphorylation. Kagami et al. revealed that IL-4 and IL-13 enhanced production of CCL26 by activation of the JAK1-STAT6-dependent pathway, but not of JAK3 [18]. Furthermore, it was reported that CCL26 production was induced in keratinocytes by IL-4 via the JAK1/2-STAT6 pathway [19].

Piperine is the major alkaloid present in fruits of the Piperaceae such as long pepper (*Piper longum*) and black pepper (*Piper nigrum*), which are used as a condiment, in perfumery and medicine. Piperine is responsible for the black pepper’s distinct biting quality such as pungent smell and taste [20,21,22]. In recent decades, various physiological effects of piperine have been reported, including antioxidant [23], anti-inflammatory [24], and anti-cancer activities [25]. In addition, piperine has been shown to ameliorate the symptoms of allergic rhinitis by inhibiting IgE levels and inflammatory cytokines in a mouse model of ovalbumin (OVA)-induced rhinitis [26], and also to improve allergic asthma via suppression of the Th2 immune response and eosinophil infiltration [27]. However, before our study, the effects of piperine on allergic dermatitis such as allergic contact dermatitis and atopic dermatitis had been unknown.

In the present study, we investigated the effects of piperine on Th2-dominant allergic dermatitis using a trimellitic anhydride (TMA)-induced AD-like mouse model [28] and dexamethasone (Dex.) as a positive control; the latter is a corticosteroid anti-inflammatory drug characterized by such effects of suppression of immune responses as inhibition of cytokine production, adhesion molecules expression, and leukocyte chemotaxis [29]. We also investigated the mechanism of action of piperine with IL-4-mediated responses in T cells and keratinocytes.

## 2. Results

### 2.1. Effects of Piperine on the Symptoms in a TMA-Induced AD-Like Mouse Model

**Figure 1:**
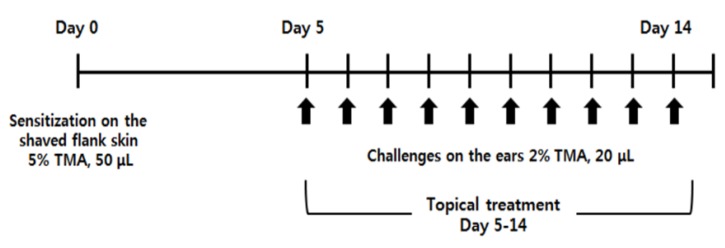
Experimental schedule of a TMA-induced AD-like mouse model. After sensitization of BALB/c mice by exposing them to 5% TMA (50 μL) on the shaved flank on Day 0, an AD-like symptom was induced on the ears of the mice by exposing them to 2% TMA (20 μL) on Days 5 to 14 daily. Piperine (2 and 4 mg/mL) or dexamethasone (10 μg/mL) was topically applied on Days 5 to 14 daily 1 h before TMA exposure.

Since TMA treatment can induce allergic dermatitis via Th2-dominant immune responses, we used a TMA-induced AD-like mouse model, induced by the method following the schedule in Figure 1, and also investigated the effects of direct topical treatment of the ears with piperine (TMA-exposed lesion).

**Figure 2:**
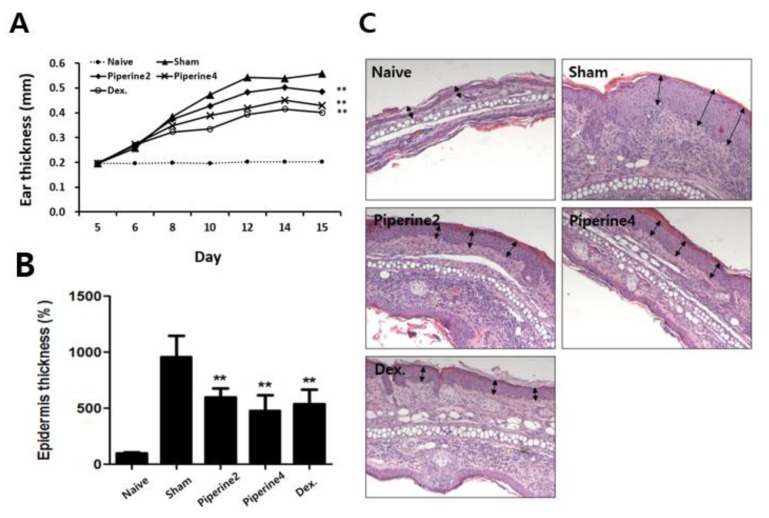
Effect of piperine on TMA-induced AD-like symptoms. (**A**) Ear thickness was measured 24 h after the TMA treatment. (**B**) The measurement of epidermal thickness is represented as the relative thickness (%) of the epidermis in the different tested groups of mice versus the sham group. (**C**) Infiltrated inflammatory cells in the ear tissues were stained with hematoxylin and eosin. Arrows indicate epidermal thickness. The results are shown as the means ± SD (Naïve, *n =* 7; Sham, Piperein2 and 4, *n =* 10; Dex., *n =* 8). Asterisks (**) indicate significant differences between the piperine-treated groups and sham groups of TMA-induced AD-like mice at *p* < 0.01.

We measured the thickness of both the ear and the epidermis as critical variables in a mouse model showing TMA-induced AD-like symptoms. As a result, ear thickness and epidermal thickness were significantly reduced by treatment with piperine (Piperine2 and Piperine4) compared with the TMA-exposed sham group (Figure 2A,B).

### 2.2. Effects of Piperine on Inflammatory and Allergic Factors in Ear Tissues

**Figure 3:**
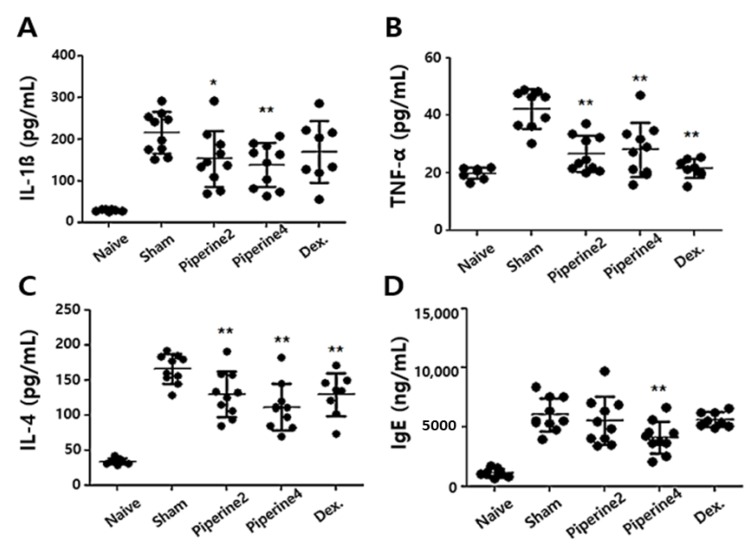
Effect of piperine on TMA-induced inflammatory cytokines and allergic responses. Production of (**A**) IL-1β, (**B**) TNF-α, and (**C**) IL-4 cytokines in the ear and (**D**) IgE levels in the serum were analyzed using ELISA. The results are shown as the means ± SD (Naïve, *n =* 7; Sham, Piperein2 and 4, *n =* 10; Dex., *n =* 8). Asterisks (*) and (**) indicate significant differences between piperine-treated groups and sham groups of TMA-induced AD-like mice at *p* < 0.05 and *p* < 0.01, respectively.

In Figure 2C, we also found that treatment with piperine could reduce the infiltration of inflammatory immune cells in ear tissues. Therefore, we investigated whether piperine suppresses inflammatory cytokines such as IL-1β and TNF-α in ear tissues. As a result, TMA treatment in the sham group increased the production of IL-1β and TNF-α, whereas the treatment with piperine in both Piperine2 and Piperine4 groups significantly inhibited the levels of IL-1β and TNF-α (Figure 3A,B).

Next, to investigate the effects of piperine on allergic immune responses, we measured typical markers of allergic immune responses-IL-4 production in ear tissues and IgE levels in sera. We confirmed that piperine treatment could reduce IL-4 production and that serum IgE levels could be increased by TMA exposure (Figure 3C,D).

### 2.3. Effects of Piperine on Th2-Associated Immune Responses Induced by TMA

Allergic immune responses are complex reactions via a number of different mechanisms occurring in various immune cells, such as allergen penetration in epithelial cells, Th2-related immune responses in Th2 cells, antibody production in B cells, and degranulation of mast cells. From these, the Th2-mediated immune response is a major reaction with regard to IL-4 production, which we investigate further.

**Figure 4:**
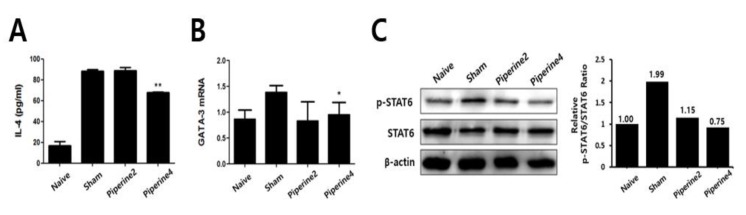
Effect of piperine on TMA-induced Th2-associated immune responses in dLNs. The dLNs were seeded to 1 × 10^6^ cells/mL and cultured in the presence of Con A (2 μg/mL) for 48 h. (**A**) The secretion of IL-4 cytokine, (**B**) the mRNA levels of GATA3, and (**C**) STAT6 phosphorylation were measured by ELISA, RT-PCR, and Western Blot assay, respectively. The results are shown as the means ± SD (*n =* 3). Asterisks (*) and (**) indicate significant differences between piperine-treated groups and sham groups of TMA-induced AD-like mice at *p* < 0.05 and *p* < 0.01, respectively.

When ears were stimulated by TMA, the dLNs of a mouse play an integral role in peripheral immune responses. Therefore, to investigate the effects of piperine on Th2-associated immune responses, we measured the IL-4 levels in dLNs cultured with Con A. We found that IL-4 production was significantly suppressed in Piperine4 over the sham group (Figure 4A). Furthermore, the mRNA levels of GATA3, a well-known transcription factor of IL-4, were significantly reduced in Piperine4. STAT6 phosphorylation, which activates GATA3 promoters [30], was also suppressed by piperine treatment (Figure 4B,C). These results demonstrated that piperine could suppress Th2-related immune responses induced by TMA through inhibition of the STAT6/GATA3/IL-4 signaling pathway in a TMA-induced AD-like mouse model.

### 2.4. Effects of Piperine on Th2 Immune Responses in Splenocytes and CD4^+^ T Cells

**Figure 5:**
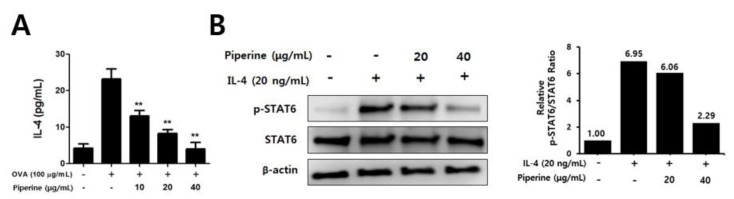
Effect of piperine treatment on IL-4 production in OVA-immunized splenocytes and on IL-4-mediated STAT6 phosphorylation in CD4^+^ T cells. (**A**) For the analysis of IL-4 cytokine production, the splenocytes isolated from OVA-immunized BALB/c mice were seeded to 5 × 10^6^ cells/mL and cultured in the presence or absence of OVA (100 μg/mL) with piperine. (**B**) To detect STAT6 phosphorylation, CD4^+^ T cells isolated from splenocytes of a BALB/c mouse were pre-treated with piperine for 1 h before IL-4 (20 ng/mL) treatment for 15 min. The results are shown as the means ± SD (*n =* 3). Asterisks (**) indicate significant differences between the piperine-treated and non-treated groups at *p* < 0.01.

Next, we investigated whether the anti-AD effects of piperine were caused by direct effect on immune cells. Since TMA acts as a hapten, it is difficult to use it as an allergen in vitro. Therefore, the direct effect of piperine was examined using splenocytes isolated from mice sensitized with OVA. As a result, we found that IL-4 production was reduced by piperine treatment in a dose-dependent manner (Figure 5A). Furthermore, we evaluated the effect of piperine on IL-4-induced STAT6 phosphorylation in CD4^+^ T cells isolated from splenocytes, and Figure 5B shows that STAT6 phosphorylation was also suppressed by piperine treatment. These results suggested that piperine could directly reduce the Th2-mediated immune response, including IL-4 production and STAT6 phosphorylation.

### 2.5. Effects of Piperine on CCR3 Cell Infiltration in Mouse Ear Tissues and IL-4-Induced STAT6 Phosphorylation in Keratinocytes

When AD progresses, the main cause is the Th2-mediated immune response, but local symptoms are derived from eosinophilic inflammatory responses through allergic mediators such as IL-4, IL-5, IL-13, and IgE. In particular, it was reported that IL-4 could induce eosinophilic inflammation in skin lesions [18]. In this study, piperine was administered topically on mouse ears, therefore, we investigated whether topical treatment of piperine directly affects keratinocytes stimulated by IL-4.

**Figure 6:**
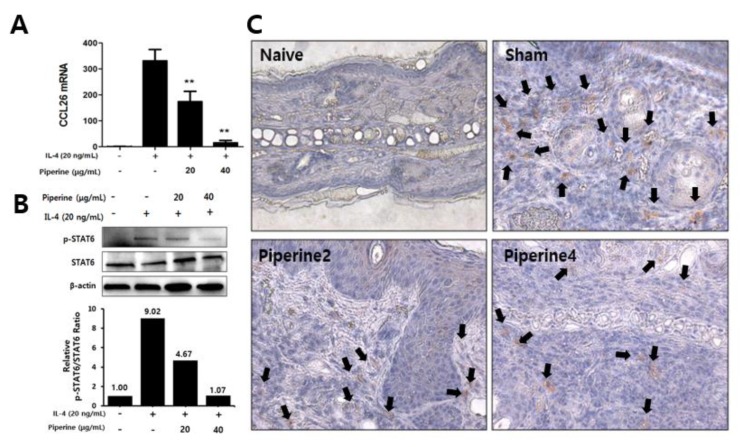
Effects of piperine on TMA-induced infiltration of CCR3^+^ cells in mouse ear tissues and IL-4-induced CCL26 mRNA expression and STAT6 phosphorylation in HaCaT cells. HaCaT cells pre-incubated with piperine were stimulated with IL-4 for 15 min and then incubated for 24 h to analyze (**A**) CCL26 mRNA expression and (**B**) STAT6 phosphorylation relatively. (**C**) Infiltration of CCR3^+^ cells into ear tissue was observed by immunohistological analysis. Black arrows indicate CCR3^+^ cells. The results are shown as the means ± SD (*n =* 3). Asterisks (**) indicate significant differences between the piperine-treated and non-treated groups at *p* < 0.01.

In Figure 4 and Figure 5, we revealed that the treatment of piperine significantly inhibited TMA- or IL-4-induced STAT6 phosphorylation in vivo and ex vivo, respectively. Therefore, we focused on the phosphorylation of STAT6 induced by IL-4 in human keratinocytes, HaCaT cells. As a result, the phosphorylation of STAT6 was increased by IL-4, and the phosphorylated STAT6 was suppressed by piperine treatment (Figure 6A).

In keratinocytes, IL-4 stimulation activated STAT6 and then produced eotaxin-3 (CCL26). The chemokines also induced infiltration of CCR3^+^ cells predominantly expressed by eosinophils [17,18]. Figure 6B shows that treatment with piperine had the effect of upregulating mRNA expression of CCL26 in keratinocytes. These results showed in vitro and in vivo correlation between chemokine expression in keratinocytes and CCR3^+^ cell infiltration in a TMA-induced AD model. The population of CCR3^+^ cells in a TMA-exposed mouse ear was reduced in Piperine2 and Piperine4 compared with the sham group (Figure 6C).

## 3. Discussion

AD is a common chronic inflammatory skin disease, which shows symptoms such as xerosis, disturbing pruritus, and eczema [31,32]. This can reduce the quality of life by causing physical and psychological problems [33,34]. The characteristics of AD are disruption of the skin barrier and immunologic dysfunction [35]. Th2-dominant immune responses result from an imbalance between the Th2 and Th1 and play a crucial role in the development of AD [36,37]. Thus, AD is generally regarded as a Th2 immune response-mediated skin inflammation in the acute phase and predominantly a Th1 immune response in the chronic phase [38]. Recently, it was reported that AD was also associated with Th17 and Th22 cells. Suárez-Fariñas et al. showed that patients with intrinsic AD have a higher immune activation of Th17 and Th22 immune axes compared to extrinsic AD [39]. Furthermore, Th22 immune responses are increased in chronic adult AD patients, whereas pediatric AD patients showed a Th1/Th2 imbalance based on Th2 dominance [1]. Although inhibition of Th17- and Th22-mediated immune responses can provide a therapeutic approach for AD, an alternative approach would be to suppress Th2-related immune responses, as 85% of AD patients show onset before the age of 5, and 70–80% of the patients show extrinsic AD [40,41].

Piperine is well known as a component of black pepper, and its anti-inflammatory effects have been proven through many studies [42,43,44,45]. In this study, our results show that treatment with piperine inhibits inflammatory cytokines, such as IL-1β and TNF-α. Both cytokines IL-1β and TNF-α can provide the signals for Langerhans cells (LCs), a subset of dendritic cells (DCs) located in the epidermis, to migrate from epidermis into dLNs in the skin [46,47,48]. Furthermore, TNF-α provokes cutaneous T cell-attracting chemokine CCL27, which leads to an infiltration of Th1 and Th2 cells into inflammatory lesions [49], and it has been known through previous studies that inflammatory cytokines such as IL-1β and TNF-α induce migration and maturation of LCs by upregulating expression of CXCR4 and CCR7 [50,51,52]. Furthermore, piperine could inhibit DCs migration and CD4^+^ T cell differentiation by inhibiting CCR7 expression and maturation of DCs [21]. In these papers, the authors suggested that piperine suppressed DCs and T cells migration in a TMA-induced AD mouse ear tissue. Therefore, we suggest that the reduction of IL-1β and TNF-α by piperine is mediated by the inhibition of DCs or migration of T cells induced by TMA treatment.

In previous reports, *Piper nigrum* and its main active component, piperine, demonstrated anti-allergic effects in OVA-induced allergic asthma and rhinitis [53,54]. The symptoms of asthma or rhinitis were ameliorated by inhibiting the degranulation of mast cells, production of inflammatory cytokines, and Th2/Th17-associated immune responses. In a previous study, we found that the *Piper nigrum* fruit extract reduced TMA-induced allergic dermatitis symptoms via suppression of STAT6 phosphorylation in splenocytes and keratinocytes [55]. However, it was not known whether piperine could improve TMA-induced AD-like symptoms. In this study, we verified that piperine could ameliorate AD symptoms by suppressing Th2-related immune responses. In addition, we topically applied piperine to mouse ears, because this method might be more effective than oral administration for the treatment of AD caused by a combination of internal and external risk factors. Despite the localized nature of the treatment, it was confirmed that Th2-associated immune responses were suppressed by piperine. Therefore, piperine might be an effective component of a cream or an ointment to alleviate AD symptoms.

According to our results, Dex. did not inhibit the production of IgE. Previous studies reported that Dex. has a different effect on T cells and B cells, which is that Dex. increases IgE production via inhibition of the expression of Bcl-6 in B cells [56]. Similarly, in some studies, Dex. treatment did not affect the IgE production [57,58]. We suggest that this result was also derived from the effect of the Dex. increasing IgE production.

Hapten, a compound smaller than 500 Da, does not act as an antigen in itself, but is recognized as an antigen inducing maturation of skin-resident DCs (LCs) by generation of hapten-protein complexes via combinations with self-proteins. Then, the antigen-bearing LCs migrate to lymph nodes and induce differentiation of antigen-specific effector Th cells by presenting antigens to naïve CD4^+^ T cells. And these effector Th cells cause an inflammatory response in the skin region by migrating from lymph nodes [59,60]. In case of TMA (hapten), it has been well known that TMA could induce allergic immune responses by provoking Th2-dominant immune responses [61], resulting in AD-like skin inflammation [28]. In the present study, we focused on Th2-related immune responses caused by TMA and confirmed that the Piperine4 group featured reduced Th2-associated immune responses such as IL-4 production, GATA3 mRNA expression, and STAT6 phosphorylation. In addition, Th2-mediated immune factors were significantly suppressed by piperine at a concentration of 4 mg/mL, but at a concentration of 2 mg/mL, the results showed low effect, irregular efficacy, and large variation. Based on these results, we suggest that the dosage of piperine should be at least 4 mg/mL for AD treatment.

**Figure 7:**
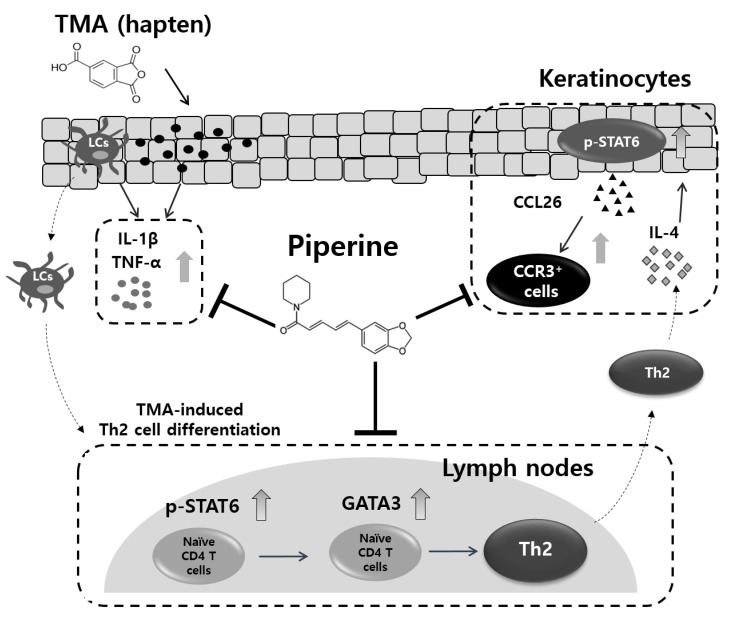
The proposed anti-AD effect of piperine in a TMA-induced AD-like mouse model.

IL-4, a representative Th2-related cytokine, induces the differentiation from naïve CD4^+^ T cells to Th2 cells via binding to the IL-4 receptor (IL-4R) [62,63,64]. Then, the phosphorylation of STAT6, the activation of GATA3 mRNA expression, and IL-4 secretion proceed sequentially. As a result, the population of Th2 cells and Th2-mediated immune responses are amplified through binding between IL-4 and IL-4R (autocrine). In this study, we revealed that treatment with piperine could inhibit Th2-mediated immune responses by suppressing the STAT6/GATA3/IL-4 signaling pathway. Since IL-4 induces production of CCL26 in keratinocytes and dermal fibroblasts in skin [65,66], we focused on CCL26 expression in HaCaT cells. As a result, these inhibitory effects of piperine could alter the production of CCL26 from keratinocytes, resulting in the suppression of infiltration by CCR3^+^ cells (Figure 7).

In particular, STAT proteins, as upstream regulators of transcriptional factors such as GATA3, T box transcription factor T-bet, retinoid-related orphan receptor (ROR)γt, and forkhead transcription factor Foxp3 in CD4^+^ T cells, are key factors to regulate differentiation of naïve CD4^+^ T cells into effector CD4^+^ T cells, such as Th1, Th2, Th17, and Treg cells [67]. Among these T cell subsets, STAT6 is related to Th2 cells, and phosphorylation of STAT6 activates Th2-mediated immune responses. In the present study, we focused on STAT6 and found that treatment with piperine suppressed the phosphorylation of STAT6 in dLNs isolated from TMA-induced mice (in vivo), CD4^+^ T cells isolated from OVA-induced mouse splenocytes (ex vivo), and HaCaT cells (in vitro). In these three models, the activation of STAT6 was detected by looking at IL-4 signals. Therefore, in a future study, we will investigate how piperine acts on STAT6 or its upstream control by Janus kinases (JAKs) and IL-4R.

In conclusion, this study demonstrates, for the first time, that piperine can ameliorate AD-like symptoms by reducing Th2-associated immune responses in a TMA-induced mouse model, and it is able to regulate STAT6 phosphorylation induced by IL-4-mediated responses in CD4+ T cells and keratinocytes. Therefore, we suggest that piperine as a candidate molecule for STAT6 control may help to improve AD symptoms.

## 4. Materials and Methods

### 4.1. Materials

Piperine, TMA, dexamethasone, and OVA (Grade VI) were purchased from Sigma-Aldrich (St. Louis, MO, USA). Inject Alum was obtained from Pierce Biotechnology (Rockford, IL, USA). The T-PER tissue protein extraction reagent was purchased from Thermo Fisher Scientific (Rockford, IL, USA). Recombinant mouse IL-4 and human IL-4 were purchased from BD Biosciences (San Diego, CA, USA). The mouse IL-4 ELISA kit was purchased from BD Biosciences (San Diego, CA, USA). The MagniSort^TM^ mouse CD4^+^ T cell enrichment kit was obtained from Invitrogen (Carlsbad, CA, USA). Antibodies specific for STAT6, β-actin, horseradish peroxidase (HRP)-conjugated goat anti-rabbit, anti-mouse antibodies were purchased from Santa Cruz (CA, USA). Antibodies for mouse phospho-STAT6 and CCR3 were obtained from Abcam (Cambridge, UK) and human phospho-STAT6 was purchased from Cell Signaling (Danvers, MA, USA). Mouse anti-CD3 and anti-CD28 antibodies were purchased from BioLegend (San Diego, CA, USA). The RNeasy^®^ Mini Kit was purchased from Qiagen (Valencia, CA, USA).

### 4.2. Animals

Five-week-old female BALB/c mice for ex vivo experiments were purchased from OrientBio Inc. (Gyeonggi, Korea). Animals were housed in an air-conditioned room (23 ± 2 °C) with a 12 h light/dark cycle and operated with in accordance with the guidelines for animal use and care of the Korea Food Research Institute (permit number: KFRI-M-16050).

### 4.3. Induction of AD-Like Symptoms by TMA

To evaluate the anti-AD effects of piperine, we used a TMA-induced AD-like mouse model by modifying the methods of previous studies [28,68]. BALB/c mice (female, 6-week-old) were divided into five groups: the naïve group (naïve, *n* = 7), the only TMA-treated group (sham, *n* = 10), the TMA and 2 mg/mL piperine-treated group (Piperine2, *n* = 10), the TMA and 4 mg/mL piperine-treated group (Piperine4, *n* = 10), and the TMA and 10 μg/mL dexamethasone-treated group (Dex., *n* = 8). The mice were sensitized with 50 μL of 5% TMA in a mixed solvent (acetone/isopropyl myristate, 4:1 v/v) on their shaved right flank on day 0, and 20 μL of 2% TMA were then repeatedly applied to the dorsal surface of both ears from day 5 to 14 daily (Figure 1). Piperine or dexamethasone (20 μL) was topically applied daily from day 5 to 14. Ear thickness was measured at 24 h after TMA treatment using a custom-built micrometer (Schering AG, Berlin, Germany). The mice were sacrificed on day 15 (Figure 1).

### 4.4. Measurement of Epidermal Thickness

Excised ears were embedded in 10% formaldehyde and cut into 6-μm-thick sections under a microscope (CM3050S Cryostat, Leica Microsystems, Wetzlar, Germany). The sections were stained with hematoxylin and 0.5% eosin (Sigma; hematoxylin and eosin [H&E] staining) to measure the epidermal thickness of the ear tissue samples. Thickness was analyzed using the Micrometrics SE Premium software.

### 4.5. Preparation of Mouse Ears for Measurement of Cytokine Levels by ELISA

Mouse ears of the AD-like model induced by TMA were mechanically homogenized in 2 mL of the T-PER tissue protein extraction reagent containing a protease inhibitor cocktail (Roche, Indianapolis, IN). The samples were then centrifuged at 25,000× *g* for 30 min at 4 °C. The concentration of total protein in the supernatant was calculated using a protein assay kit (Bio-Rad Laboratories, Hercules, CA, USA) and diluted to 1 mg/mL with a phosphate-buffered saline containing Tween 20 (0.5%).

### 4.6. Culture of Draining Lymph Nodes

The draining lymph nodes (dLNs) of the mice were cultured for measurement of IL-4 cytokine production. Briefly, dLNs isolated from mice were separated into single cells and cultured with 2 μg/mL concanavalin A (Con A, Sigma-Aldrich) in the RPMI 1640 media containing 10% FBS, 100 U/mL penicillin, and 100 mg/mL streptomycin. The cells were incubated to a density of 1 × 10^6^ cells/mL for 48 h in a humidified incubator with 5% (*v*/*v*) CO_2_ and 95% (*v*/*v*) air, and a culture supernatant was used for the ELISA assay.

### 4.7. Induction of Th2-Dominant Immune Responses and Cell Culture

BALB/c mice (female, 6-week-old) were sensitized with an intraperitoneal injection of 10 μg OVA adsorbed on 1 mg of alum on days 0 and 14. The mice were sacrificed on day 28. Splenocytes isolated from the mice were cultured to 5 × 10^6^ cells/mL in the RPMI 1640 medium containing 10% fetal bovine serum, 100 U/mL penicillin, and 100 mg/mL streptomycin. The cells were incubated at 37 °C for 72 h in a humidified incubator with 5% (*v*/*v*) CO_2_ and 95% (*v*/*v*) air in the presence or absence of OVA (100 μg/mL) and piperine (20 and 40 μg/mL).

### 4.8. Measurement of Cytokine Levels by ELISA

Cytokine levels in the ear supernatant or cell culture medium were measured using ELISA kits for IL-4, IL-1β, and TNF-α. IgE in serum was also detected with an IgE ELISA. All ELISA analyses were performed according to the manufacturers’ instructions.

### 4.9. CD4^+^ T Cell Isolation and Induction of STAT6 Phosphorylation Treated by IL-4

CD4^+^ T cells were isolated from BALB/c mouse splenocytes with the Magnisort^TM^ mouse enrichment kit (Invitrogen, Carlsbad, CA, USA). Purified CD4^+^ T cells were cultured with anti-CD3 antibody (1 μg/mL) and an anti-CD28 antibody (1 μg/mL) for 48 h and starved for 24 h in serum-free media. CD4^+^ T cells were centrifuged at 1300 rpm for 5 min and the supernatant was removed. Next, CD4^+^ T cells were pre-incubated with piperine (20 and 40 μg/mL) for 1 h before treatment of IL-4 (20 ng/mL) for 15 min.

### 4.10. Induction of STAT6 Phosphorylation and CCL26 mRNA in HaCaT Cells Treated with IL-4

Immortalized human epidermal keratinocytes (HaCaT cells) were cultured at 37 °C in a Dulbecco’s modified Eagle’s medium (DMEM) containing 10% fetal bovine serum, 100 U/mL penicillin, and 100 mg/mL streptomycin in a 5% CO_2_ humidified incubator. The HaCaT cells were pre-incubated with piperine for 1 h before stimulation with IL-4 (20 ng/mL) for 15 min to detect STAT6 phosphorylation, and cultured with IL-4 (20 ng/mL) for 24 h to analyze the CCL26 mRNA expression.

### 4.11. Western Blotting Analysis

Protein concentration was determined using a dye-binding protein assay kit (Bio-Rad Laboratories, Berkeley, CA, USA) following the instructions in the manufacturer’s manual. Lysate protein was subjected to 10% sodium dodecyl sulfate-polyacrylamide gel electrophoresis (SDS-PAGE) and transferred to a polyvinylidene difluoride (PVDF) membrane from Amersham Pharmacia Biotech, (Buchinghamshire, UK). After the transfer, the membranes were incubated with specific primary antibodies at 4 °C overnight. Protein bands were visualized using an AE-9300 Ez-Capture MG (ATTO Corporation, Tokyo, Japan) after hybridization with a horseradish peroxidase (HRP)-conjugated secondary antibody.

### 4.12. Immunohistological Analysis

Paraffin-embedded mouse ear tissue sections were cut to a thickness of 5 μm using a microtome and air-dried at room temperature overnight. Paraffin sections were de-paraffinized using xylene and hydrated in descending grades of ethanol in distilled water; after that, antigen retrieval was performed by heating samples to 95–100 °C in 10 mM citrate buffer at pH 6 for 10 min. For immunofluorescence, sections of mouse ear were permeabilized and blocked at room temperature by incubation in the PBS containing 0.02% Tween 20 and 1% BSA for 1 h. Incubation with an anti-CCR3 antibody diluted in the PBS containing 3% BSA was conducted at 4 °C overnight. A secondary antibody and staining solution was applied using a Singalstain^®^ DAB substrate kit (Cell Signaling Tech.). The rabbit IgG antibody was added at a 1:1000 dilution for 1 h at room temperature. DAPI (1:10,000) was used as a nuclear stain. Sections were examined and photographed using a Nikon ECLIPSE Ti-s microscope (Nikon, Tokyo, Japan). Images were processed using the Photoshop (Adobe) software.

### 4.13. Quantitative RT-PCR Analysis and mRNA Isolation

Total RNA was recovered and purified using an RNeasy Mini Kit (Qiagen, Hilden, Germany) according to the manufacturer’s instructions. cDNA was synthesized using a QuantiTect Reverse Transcription Kit (Qiagen, Hilden, Germany). First-strand cDNA was prepared from 1 μg of total RNA. The samples were subjected to real-time PCR using a SYBR Green master mix on a Rotor-Gene Q 2plex System (Qiagen, Hilden, Germany). The gene expression levels were normalized to those of GAPDH. Relative gene expression changes were calculated using the 2-delta CT method and reported as a fold change over the control samples. The sequence of primers was as follows: CCL26, (forward) 5′-AAC TCC GAA ACA ATT GTG ACT CAG CTG-3′ and (reverse) 5′-GTA ACT CTG GGA GGA AAC ACC CTC TCC-3′; GAPDH, (forward) 5′-TGT GTC CGT CGT GGA TCT GA-3′ and (reverse) 5′-CCT GCT TCA CCA CCT TCT TGA-3′. The primers used in this experiment were purchased from Bioneer (Daejeon, Korea).

### 4.14. Statistical Analysis

The data are expressed as the means ± standard deviations (SD), with significant differences determined using one-way ANOVA (analysis of variance). A probability value of *p* < 0.05 was used as the minimum threshold for statistical significance.
